# A Review of the Rumen Microbiota and the Different Molecular Techniques Used to Identify Microorganisms Found in the Rumen Fluid of Ruminants

**DOI:** 10.3390/ani14101448

**Published:** 2024-05-13

**Authors:** Éder Bruno Rebelo da Silva, Jamile Andréa Rodrigues da Silva, Welligton Conceição da Silva, Tatiane Silva Belo, Carlos Eduardo Lima Sousa, Maria Roseane Pereira dos Santos, Kedson Alessandri Lobo Neves, Thomaz Cyro Guimarães de Carvalho Rodrigues, Raimundo Nonato Colares Camargo-Júnior, José de Brito Lourenço-Júnior

**Affiliations:** 1 Postgraduate Program in Animal Science (PPGCAN), Institute of Veterinary Medicine, Federal University of Para (UFPA), Castanhal 68746-360, Brazil; welligton.medvet@gmail.com (W.C.d.S.); thomazguimaraes@yahoo.com.br (T.C.G.d.C.R.); camargojunior@gmail.com (R.N.C.C.-J.); joselourencojr@yahoo.com.br (J.d.B.L.-J.); 2 Institute of Animal Health and Production, Federal Rural University of the Amazônia (UFRA), Belem 66077-830, Brazil; jamileandrea@yahoo.com.br; 3 Department of Veterinary Medicine, University Center of the Amazon (UNAMA), Santarém 68010-200, Brazil; tatianebelovet@gmail.com (T.S.B.); cadu34.medvet@gmail.com (C.E.L.S.); 4 Institute of Engineering and Geosciences, Federal University of Western Pará (UFOPA), Santarém 68040-255, Brazil; roseanemaria022@gmail.com; 5 Institute of Animal Science, Federal University of Western Pará (UFOPA), Santarém 68040-255, Brazil; kedson_neves@hotmail.com

**Keywords:** microbial interactions, environmental variations, nutrition improvement

## Abstract

**Simple Summary:**

Environmental factors like climate, diet, and farming methods significantly affect microbial activity and composition. Understanding these adjustments is crucial for improving ruminant nutrition and production. This review summarizes research on rumen microbiota and molecular methods for microbial identification in ruminant rumen fluid. Analyzing rumen microbiology in various ruminants is challenging due to environmental and nutritional influences on microbial interactions. Key genera include *Entodinium* spp. and *Aspergillus* spp. for protozoa and fungi, respectively, with *Fibrobacter* spp. prevalent among bacteria. Major techniques involve DNA extraction, amplification, and sequencing. In conclusion, this review highlights the knowledge in the literature about rumen microorganisms and the associated molecular approaches.

**Abstract:**

Variations in environments, including climate, diet, and agricultural practices, significantly impact the composition and microbial activity. A profound understanding of these adaptations allows for the improvement of nutrition and ruminant production. Therefore, this review aims to compile data from the literature on the rumen microbiota and molecular techniques for identifying the different types of microorganisms from the rumen fluid of ruminants. Analyzing the literature on rumen microbiology in different ruminants is complex due to microbial interactions, influenced by the environment and nutrition of these animals. In addition, it is worth noting that the genera of protozoa and fungi most evident in the studies used in this review on the microbiology of rumen fluid were *Entodinium* spp. and *Aspergillus* spp., respectively, and *Fibrobacter* spp. for bacteria. About the techniques used, it can be seen that DNA extraction, amplification, and sequencing were the most cited in the studies evaluated. Therefore, this review describes what is present in the literature and provides an overview of the main microbial agents in the rumen and the molecular techniques used.

## 1. Introduction

Ruminant breeds in different production systems tend to exhibit the influence of environmental conditions on their means of productivity, mainly in tropical and subtropical regions, where there is the predominance of high temperatures throughout the year [1,2,3]. In this context, metabolic changes in the host can occur in production environments, favoring changes in the rumen microbiota and interfering with nutrient absorption [4,5,6,7,8].

The multicavitary stomach, consisting of the rumen, reticulum, omasum, and abomasum are important morphological structures of ruminants that may undergo alteration according to the established diet [9]. The structure of the stomach allows the feeding process to have a well-defined grazing cycle. Ruminants spend between a third and a half of their time grazing on as much food as possible, storing all this consumption in the first of these cavities—the rumen [10].

Microorganisms are responsible for carrying out various processes in digestive action, with the aim of improving the animal’s nutritional performance. The rumen microbiota is made up of different microorganisms depending on the region where the animal is reared and the diet used [11,12].

In research that adopted different techniques and ruminant breeds, the structure of the ruminal environment was observed, making it possible to identify the microbiome of rumen [13]. The fungi, bacteria, archaea, and protists common in the ruminal environment are producers of enzymes that break down the ingested plant material, producing vitamins, amino acids, and volatile fatty acids, the main primary source of energy for ruminants [14].

Understanding the integration of taxonomic and functional data on microbial populations and their interactions with rumen management, with the performance and metabolism of cattle, can favor gains in the medium and long term [15,16]. In addition, to propose improvements in ruminant breeding, it is necessary to understand how the nutritional process of these animals works, which is highly dependent and carried out by the symbiotic relationship with different microorganisms [17].

In this context, understanding the rumen microbiota is essential due to its economic importance in livestock farming, as well as providing subsidies for improvements in production, since the microbial pattern of the rumen can be associated with weight gain, feed efficiency, residual feed intake, dry matter intake in beef cattle, milk production, milk quality, reproductive efficiency, and immunology in dairy cattle.

Despite the progress made in understanding the rumen microbiota and the molecular techniques used to identify microorganisms found in the rumen fluid of ruminants, there are still gaps and challenges to be addressed. One of the main ones is the need for a better understanding of the interaction between the different microorganisms in the rumen environment and how these interactions affect animal health and performance. In addition, the variability of the rumen microbiota between different species of ruminants and in different environments has yet to be fully elucidated.

Another aspect to consider is the identification and characterization of new species of microorganisms in the rumen fluid. The improvement and standardization of molecular techniques are also necessary to ensure an accurate and consistent assessment of the rumen microbiota. Therefore, the aim of this review is to compile data from the literature on the rumen microbiota and molecular techniques to identify the different types of microorganisms found in the rumen fluid of ruminants, thus providing support for future research.

## 2. Material and Methods

For the development of this review, a bibliographic survey was conducted in databases Web of Science, Scopus, and PubMed, which are scientific databases capable of storing high-quality articles. The following keywords were used: “rumen microorganisms”, “ruminants rumen microbiota”, “carbohydrates in ruminants diet”, and “molecular tests of ruminal fluids”. The inclusion criteria were full text, written in English, Portuguese, and Spanish, and there was a discussion on the characterization of ruminant microbiota. The articles were excluded when they did not fit into the purpose of the study.

No time or geographical limits were used during the searches. All the literature published up to the end of the database searches was considered. A total of 94 references were cited in this review. This methodology was also adopted in the study by Mota-Rojas et al. [18] and Camargo-Júnior et al. [19].

**Figure 1:**
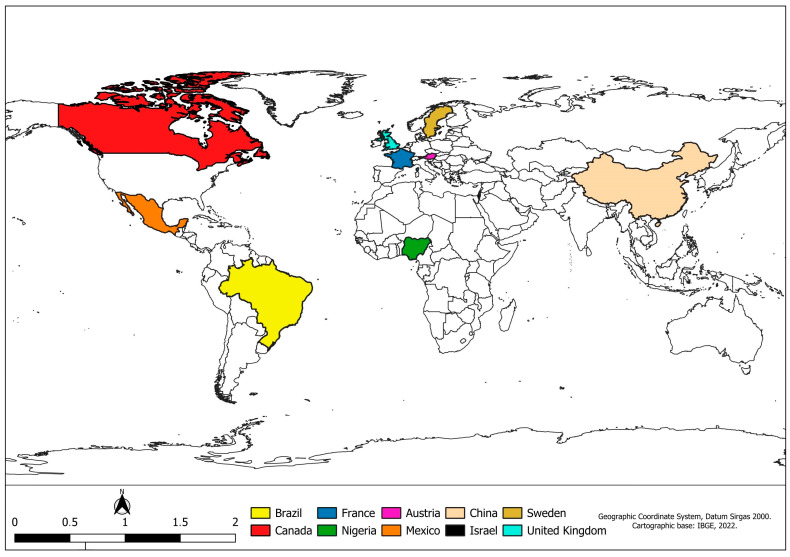
Map of the countries found in the database platform searches and used in this review.

Figure 1 shows the countries that have carried out studies on the subject and are included in this review.

Table 1 shows the data obtained during the bibliographic survey, using the pre-defined keywords mentioned above, considering the year, authors, study title, species, breed, sample, and diet. This table does not include review articles, only experimental studies.

Table 2 shows the authors who have carried out research using different techniques for identifying microorganisms.

## 3. Literature Review

### 3.1. Rumen Microbiota

Ruminants are considered the most important taxonomic category, among the species of animals with the greatest diversity in food production, such as meat and milk. They are herbivorous animals capable of transforming the energy stored in plants into digestible food products, since the digestive system has the ability to deteriorate and absorb significant amounts of plant material, through the anaerobic compartment, located in the previous portion of the gastrointestinal tract of ruminants [55].

In this sense, the diet can determine the characteristics of the ruminal environment, where the temperature is regulated by metabolism, remaining between 30 and 42 °C, with pH in the range of 5 to 7. Thus, the content of the rumen is formed by liquid and solid material, as well as by gases formed during the fermentation process of the fibers [23,35,36].

The vegetable material present in the food of ruminants is decomposed and fermented by a series of microorganisms housed in the ruminant, responsible for providing energy and protein to the animal, in the form of short-chain fatty acids (AGCC) including acetic, propionic, and butyric, as well as by the presence of microbial protein and vitamins, which are being used as precedents of fat (acetic and butyric) or propionic glucose, providing a symbiotic relationship with the host [43,56,57].

The microbiota is composed of a set of thousands of species of microorganisms, consisting of bacteria, archaea, fungi, and protozoa [39,58]. At significant values, the bacteria represent the largest and most important ruminal microbial community, consisting of approximately 1.010 bacteria, 10^7^ protozoa, 10^6^ fungi per mL of rumen content [44], and 10^7^–10^8^ cells per gram of ruminal content of methanogenic archaea [37,59].

As noted in the literature, different types of bacteria present in the ruminant are noted with known metabolic properties, with *Fibrobacter succinogenes* and *Ruminococcus flavefaciens* being the main bacteria responsible for the degradation of fiber foods, and *Prevotella ruminicola*, *Eubacterium ruminantium*, *Anaerovibrio lipolytica*, and *Streptococcus bovis* responsible for non-fibrous foods [26,40,60].

These species are categorized according to the substrate used and the products of their fermentation, classified into structural and non-structural, lipolytic, proteolytic, and lactic carbohydrate fermenting bacteria [61,62,63]. Consequently, bacteria, besides being present in ruminal fermentation and providing energy to ruminants, are also the largest producers of enteric methane, the natural gas derived from the fermenting of food consumed by animals by means of methanogenic archaea that use free hydrogen (H_2_) and carbon dioxide (CO_2_) as substrates [30,41,64].

In view of this, the end products of fermentation metabolize the hydrogen gas to reduce CO_2_ and form the methane gas through methanogenesis, a method responsible for the synthesis of energy produced from adenosine triphosphate (ATP) to the microorganism. In this way, the production of methane keeps the concentrations of hydrogen low, allowing the proliferation of other species in the rumen and enabling efficient fermentation [33,65,66].

However, it is estimated that ruminants are responsible for the production of about 14% of methane gas, one of the factors that may contribute to the establishment of the greenhouse effect [42,58]. The main suppliers of methane-producing microorganisms in the rumen are the methanogenic archaea, which are anaerobic beings capable of using different types of substrates to produce methane, and the presence of bacteria and ciliated protozoa, with the highest percentage of *Methanobrevibacter* spp. (63.2%), followed by *Methanosphaera* spp. (9.8%), *Methanomicrobium* spp. (7.7%), *Thermoplasma* spp. (7.4%) and *Methanobacterium* spp. (1.2%) [27,31,67].

### 3.2. Carbohydrates in Ruminant Diet

Structural carbohydrates found in plant tissues and formed by plant cell walls are called fibers. In relation to nutritional value, these macromolecules are known as fibrous carbohydrates [68]. Carbohydrates in the diet of ruminants play an extremely important role in the development of the gastrointestinal tract and in the health and nutrition of these animals [69]. It should also be noted that a diet rich in concentrates increases the risk of ruminal acidosis in ruminants [36].

Carbohydrates are divided into structural (SCs)/fibrous contained in the cell wall and non-structural (NSCs)/non-fibrous present in the cell content [70]. The presence of structural or non-structural carbohydrates in ruminant diets is indispensable because, in addition to being the main source of energy due to the presence of starch and fiber, they help regulate the fermentation process in the rumen [71].

Thus, rumen bacteria can be classified using fermentation characteristics. The bacteria that decompose structural carbohydrates (cellulose and hemicellulose) are known as cellulolytics that fragment cellulose through enzymatic complexes (cellulases). Bacteria that break down non-structural carbohydrates (starch, pectin, and sugars) are characterized as amylolytic and pectinolytic, and interact in the production of amylases and pectinases, respectively. Thus, the amount of fibrous and non-fibrous carbohydrates becomes indispensable to keep the ruminal environment stable [72].

## 4. Microorganisms Observed in the Ruminal Microbiota

### 4.1. Protozoa

The protozoa are microorganisms of great importance in the ruminal microbiota [73], unicellular, anaerobic, and non-pathogenic, with a size between 20 and 200 μm and an approximate density of 104 protozoa per milliliter in the ruminal content. Their importance is due during the fermentation process, as they participate in the digestion of most of the food components, with emphasis on the digestibility of fibers, and can be responsible for up to 34% of the degradation of fibrous material [74].

The protozoa located in the rumen are responsible for several interconnected functions, mainly when it comes to nutrient metabolism. The ruminant fauna account for 40 to 60% of the biomass, highlighting the class of the Ciliates, since the main identified species are the following: *Isotricha intestinalis*, *Isotrycha prostoma*, *Ophryoscolex purkynjei*, *Ophryoscolex inermis*, *Entodinium bursa*, *Entodinium dentatum*, *Entodium caudatum*, *Buetschlia* spp., and *Dasytricha* spp. [75].

These ciliated beings are present in a density of 10^4^ to 10^6^ cells/mL of ruminal fluid, responsible for 30 to 40% of the total fiber consumption. Protozoa are quite active in the breakdown of lipids and tend to make hydrogen available through their hydrosomes [67].

In the case of the fermentative substrate, protozoa can be defined as sugar consumers, deteriorating starch, and hydrolyzing complex carbohydrates (lignin and cellulose). However, sugars and starch are transformed into fermentative substrates that are absorbed, causing a buffer effect on the rumen, and reducing the presence of ruminal acidosis in diets rich in grains or sugars [68].

**Figure 2:**
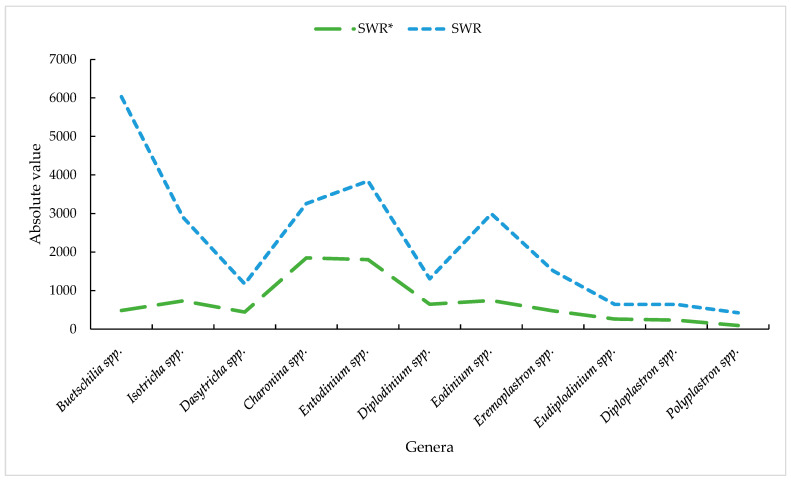
Ruminal protozoa of bulls raised with and without bulk. Note: SWR*—steers with roughage; SWR—steers without roughage. Adapted from Nigri et al. [34].

Nigri et al. [34] carried out a study with 50 Nelore cattle with the aim of assessing the population of protozoa in the rumen. The animals were divided into two groups, identified as G1 and G2. The groups had different nutrition, with group 1 or G1 being fed *Brachiaria* spp. and mineral supplement and group 2 or G2 being fed a diet consisting of vitamin, protein, and mineral concentrate with corn grains. In this study, the authors identified 17 genera of protozoa, of which *Dasytrichia* spp., *Charonina* spp., *Eudiplodinium* spp., *Entodinium* spp., and *Diplodinium* spp. were more common in G1. The authors suggest that feeding *Brachiaria* spp. forage favors the presence of these genera in the rumen. On the other hand, G2 had different genera, with a prevalence of *Buetschilia* spp., *Isotricha* spp., *Eodinium* spp., *Polyplastron* spp., *Elytroplastron* spp., *Metadinium* spp., and *Enoploplastron* spp. varying due to the consumption of concentrate in the diet, highlighting the extremely important impact of food consumption on the composition of the rumen microbiota, influencing both animal health and the fermentation process within the rumen (Figure 2).

**Figure 3:**
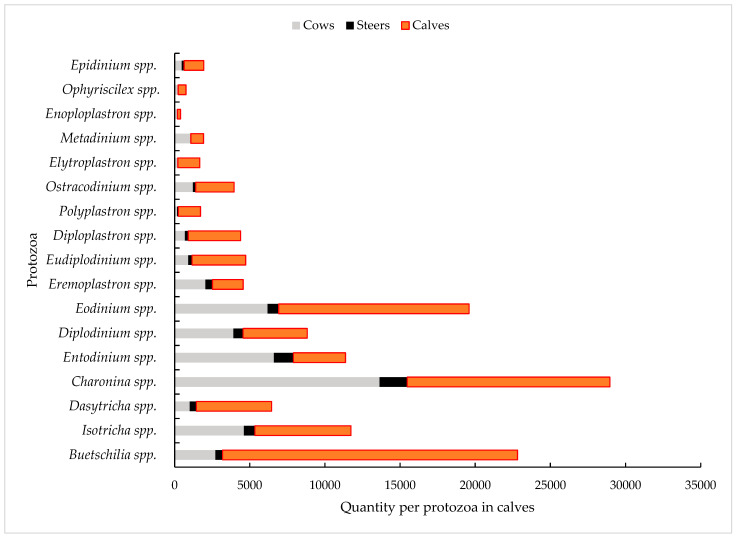
Variety of protozoa identified in the rumen of mixed-race Nelore cattle at different ages. Adapted from Duarte et al. [38].

The study by Duarte et al. [38] quantified the protozoa present in the rumen of mixed-breed Nelore raised in the state of Minas Gerais, comprising 36 bulls, 34 cows, and 30 calves of varying ages. The study revealed 135,000 protozoa with various genera identified, with a predominance of the genus *Charonina* spp. The data highlight the importance of analyzing the diversity and complexity of protozoa involved in the metabolism and formation of the rumen microbiome. This diversity of genera is related to the fermentation and digestibility process and contributes to the degradation of dietary fibers, which can be influenced by food consumption and the physiological state of the animals (Figure 3).

The same authors also documented higher incidences of *Entodinium* spp. in adult cattle [38]. The same was observed in the study of Abrar et al. [32], in which a considerable concentration of this eukaryote (80%) was noted in adult cattle of the Holstein and Wagyu breeds fed with olive straw and commercial concentrated.

Martinele et al. [20] also identified this ciliate at significant concentrations (79%) in Dutch–Zebul cross cows that received varying proportions of elephant herd (60–100%). In addition, Ríspoli et al. [22] reported an incidence of 85% of *Entodinium* spp. in Holstein cows fed with 50% maize silage and 50% concentrated maize (maize in grains and soy flour).

According to Newbold et al. [76], the genus *Entodinium* spp. plays a key role in the renewal of bacterial proteins in the rumen. A variety of genera of protozoa in the ruminal environment is intrinsically associated with the type of diet offered [77,78]. Research indicates that the predominance of concentrates in the diet favors the increased concentration of these eukaryotes [79].

Tymensen et al. [25] found a variety of ruminal protozoa in calves fed with hay or grain silage. The genus *Entodinium* spp. was most abundant in cattle fed with grain silage when microscopic methods were used. By contrast, the heterogeneity of protozoa in the rumen was greater for hay-fed animals, showing greater stability.

In addition, climate patterns established in different regions with well-defined seasons can favor a wide diversity of protozoan populations. The same study showed that in *Bos indicus*, there was a greater number of microorganisms in wet seasons, which can be explained by the improvement in the nutritional quality of grasses used in animal feeding [28].

Other authors also observed the influence of the diet on the concentration of microorganisms in periods of drought when the quality of the available pasture is lower, promoting a reduction in the concentrations of protozoa. In breeding cows, the presence of large amounts of identified protozoa corresponds to the *Ophyroscolecidae* family, such as the genera *Epidinium* spp., *Polyplastron* spp., and *Eudiplodinium* spp. [28,80].

### 4.2. Bacteria

**Figure 4:**
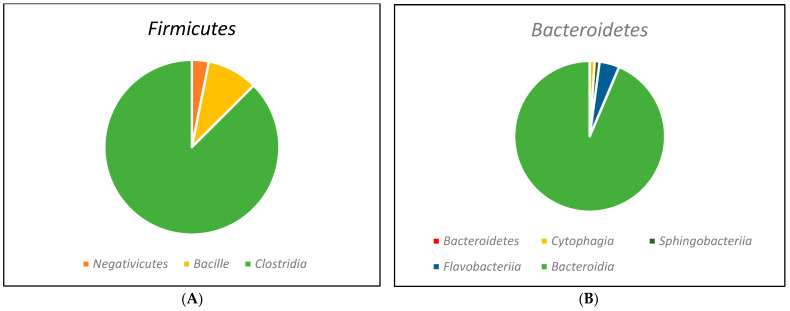

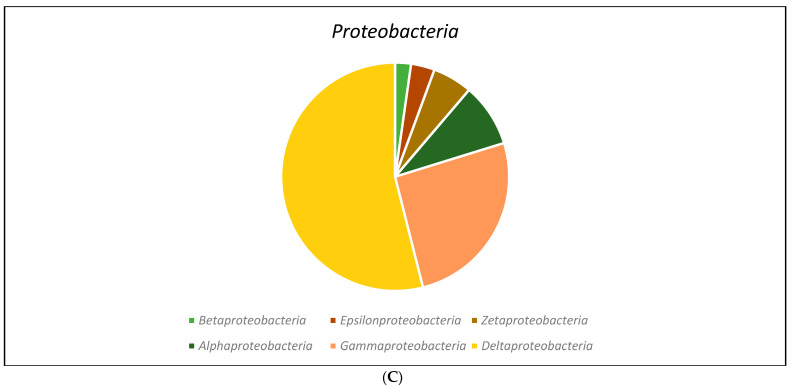
Class-level taxonomic affiliations for reads generated from 16S rRNA fragment sequences of bovine rumen contents using the Illumina platform. (**A**) Taxonomic classification of the phylum *Firmicutes*; (**B**) taxonomic classification of the phylum Bacteroidetes; (**C**) taxonomic classification of the phylum *Proteobacteria*. Adapted from Jesus et al. [39].

Figure 4 shows the taxonomic classification of the class of the seven main genera of bacteria identified in the rumen contents of cattle. The Firmicutes phylum presented three classes (Figure 4A) and Bacteroidetes revealed the presence of four classes each (Figure 4B), while the Proteobacteria phylum presented six distinct classes (Figure 4C). However, in this same study, the phyla *Spirochaetes*, *Fibrobacteres*, *Tenericutes,* and *Actinobacteria* were found to have only one class for each phylum. This detailed taxonomic approach provides relevant data on the diversity and structure of the microbiome, and the presence of multiple classes found in the phyla suggests the importance of metabolic and adaptive pathways aimed at rumen fermentation and nutrient metabolism, playing a very important role in rumen function. It should also be noted that understanding the relationships between microorganisms and their hosts has a direct impact on their health, physiology, nutrition, and animal productivity.

**Figure 5:**
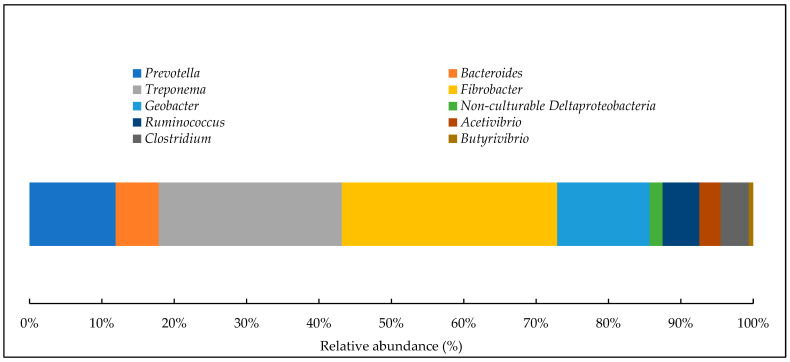
Gender-level taxonomic affiliations for readings generated from the 16S rRNA fragment sequences of bovine ruminal content using the Illumina platform. Adapted from Jesus et al. [39].

Figure 5 shows the taxonomic classification of the main bacterial genera found in the rumen contents of ruminants. The most abundant genera were *Fibrobacter* and Treponema, followed by *Geobacter* and *Prevotella*. This analysis provides a detailed view of the bacterial diversity found in the rumen of these animals, especially in relation to fiber degradation and fatty acid production. All of these genera are essential for helping with metabolism and determining specific functions within the rumen environment.

**Figure 6:**
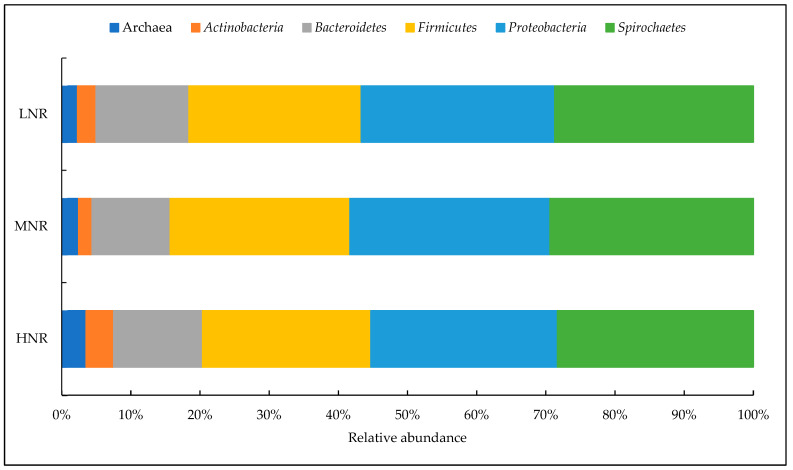
Ruminal and archaea bacteria identified in cattle managed under different levels of nitrogen. LNR—low nitrogen utilization. MNR—medium nitrogen utilization. HNR —high nitrogen utilization. Adapted from Alves et al. [46].

Alves et al. [46] conducted a study in São Paulo at the Universidade Estadual Paulista (UNESP) with eight cattle, and identified different species of bacteria and archaea, as shown in Figure 6, from the use of three different types of diet offered to the animals, with sugar cane used as the bulk in this study and a concentrate with different protein sources. Sugar cane has a high fiber content that has an impact on the composition of the rumen microbiota of these animals, as well as the offer of different protein sources, which favored the appearance of other genera, demonstrating the importance of knowing and understanding the diversity and response of the microbial composition in relation to the diet, in order to improve the nutrition and productivity of the animals.

Jesus et al. [81] conducted a study seeking to characterize bacterial diversity through partial sequencing of a total 16S rDNA and electronic scanning microscopy (ESM) of the ruminal microbiome, and identified a bacterium, fungal, and protozoan diversity. The authors point to bacteria in the genera *Bacteroides* spp., *Prevotella* spp., *Ruminobacter* spp., *Fibrobacter* spp., and *Methanobrevibacter* spp. (spores) and in the bunch of *Butyrovibrio* spp., *Selenomonas* spp., and *Lachnospira* spp., as well as *Ruminococcus* spp., *Streptococcus* spp., and *Megasphaera* spp.

Lima et al. [47] conducted a study aiming to characterize the indices of richness and diversity of the bacterial community of the rumen. For this, four Nelores cattle were used, which received the following four diets: T1—no additive (CON); T2—inclusion of 90 g of 33 sodium bicarbonate (BIC); T3—inclusion of 90 g of *Lithothamni-a calcareum* (L90); and T4—34 incl. of 45 g of *Lithehamnium calcareum* (L45). The authors pointed out that the greatest wealth was in the genera *Prevotella* spp. (48%, 723 OTUs), followed by *Treponema* spp. (20%, 300 OTU’s%), *Fibrobacter* spp. (4%, 55 OTU’s), *Butyrivibrio* spp. (4%, 54 OTU), *Anaeroplasma* spp. (3%, 48 OTU), and *Ruminococcus* spp. (3%, 44 OTU). The dominant genera were *Prevotella* spp. (77.84%), *Fibrobacter* spp. 331 (11.37%), *Treponema* spp. (4.68%), *Rumunobacter* spp. (1.26%), *Alistipes* spp. (1.09%), *Elusimicrobium* spp. 332 (0.83%), *Ruminococcus* spp. (0.67%), and *Anaeroplasma* spp. (0.65%).

Freitas et al. [45] conducted a study with 17 samples of cows, approximately 36 months old, provided by the Department of Diagnostics and Agriculture Research, in São Gabriel, Rio Grande do Sul, Brazil, and found 23 microbial phylos, including 19 genera of bacteria and four fungi. Differential analyses in relation to abundance showed that the distribution of phylum between average weight gain (AWG) among groups was very large, being similar in bacteria and fungi.

### 4.3. Fungi

The presence of fungi in the rumen is little in the scientific literature. A study conducted in the state of Minas Gerais, Brazil, evaluated the population of anaerobic fungi in the rumen of cows and calves of the Dutch breed fed with different types of bulk. The genera of fungi *Aspergillus* spp. (56%), *Rhizophus* spp. (12.8%), *Trichophyton* spp. (8,5%), *Paecilomyces* spp. (7.1%), and *Scedosporium* spp. (6.3%) were the most evidenced in this study [29].

Brewer and Taylor [82], in a pioneering study on the fungi present in the rumen fluid of cattle reared in an extensive system in England, observed the presence of the genera *Aspergillus fumigatus* and *Sporomia* spp. Another study also highlighted the significant presence of this fungus in rumen fluid samples from cows [21].

In the same study, the genera *Fusarium* spp., *Penicillium* spp., *Aspergillus* spp., and *Mucor* spp. were the most prevalent. In contrast, in samples that were specifically isolated from ruminal juice from cows, the genus *Mucor* spp. was the most prevalent, followed by *Aspergillus flavus* [21].

The findings corroborate the study by Flipphi et al. [83] that identified the micromorphology of isolated fungi, evidencing that the genus *Aspergillus* spp. was the most common in confined cattle. The predominance of this genus can be explained due to its great versatility and efficiency in the catabolism of soluble carbon sources.

Almeida et al. [24] reported the presence of the yeasts of *Candida krusei*, in the rumen of dairy cows bred in the north of Minas Gerais, Brazil, being an important agent of mycoses that affect both animals and humans. The study also highlighted the relevant ecological and pathogenic role of this and other yeasts in improving the productivity and health of these animals.

The question of the contribution of anaerobic fungi to microbial biomass in the rumen continues to arouse debate. While the flagellated zoospores are easily visible in the ruminal fluid, the less obvious manifestation occurs in the vegetative growth of the rhizoids on and over plant material. Chitin analysis and assessment of the abundance of rRNA transcripts, as described by Huws et al. [58], suggest that anaerobic fungi comprise 10% to 20% of the ruminal microbiome, which is considered of crucial importance in fiber degradation, especially when ruminants are fed low-quality feed, as highlighted by Krause et al. [84].

Edwards et al. [85] highlighted that the binding of fungi and archaea in the rumen is an activity that enhances the fungal activity and the production of methane, with six of the following genera (often more): the monocentric—*Neocallimastix* spp., *Caecomyces* spp., and *Piromyces* spp.; and the polycentrical—*Anaeromyces* spp., *Orpinomyces* spp., and *Cyllamyces* spp.; however, other genera could be more described, resulting in various genomes.

## 5. The Use of Molecular Techniques in the Characterization of the Ruminal Microbiome

The identification of ruminal microorganisms was analyzed by means of classic cell culture techniques, such as the isolation of bacteria, which restricted knowledge of microbial diversity [51]. In the current scenario, we can observe the rapid evolution in the study of microbial ecosystems, with the insertion of new molecular techniques, which help in the identification of a greater proportion of microorganisms, including sequences of those not yet isolated.

These methods are used to detect certain organisms or amplified taxonomic groups, such as evidence of target regions of the ribosomal RNA molecule (RNA), with different levels of variability since the bacterial genome is considerably stable and is not affected by growth conditions. For the categorization of the ruminal system, one of the most modern techniques is employed, called new generation sequencing (NGS), developed mainly for the mapping of the human genome [86].

Other methods can be used within the NGS, favoring a more advantageous platform over the previous techniques, such as the Sanger, which usually presents results in months or years, in contrast with these methods that are completed in days or weeks, thus possessing advantages such as high speed, high yield, and low cost–benefit ratio in the sequencing of samples [87].

Another benefit to be acquired is the reduction of DNA into single, amplified fragments, rather than generating clones. Genotyping sequencing (GBS), also referred to as restrictive enzyme representation sequencing, is a technique that uses particles to reduce the complexity of the genome by digesting genomic DNA with the use of restrictive enzymes [54].

Genotyping is widely used for the best performance of plants, cattle, and aquatic beings, as well as population diversity studies, such as conservation and genomic selection projects in gene mapping and genome studies in various varieties of crops [88]. The parallel study of a community of microorganisms, instituted by means of the sequencing of DNA fragments and series assembly of the original molecules, i.e., the direct analysis of DNA from a set of microorganisms, is called metagenomics [52].

In a general context, the analyses of the metagenomic approach include four main stages: sample preparation; DNA extraction; sequencing of genetic material; and analysis using bioinformatics tools. In theory, any type of sample can be analyzed using a metagenomic approach. About sample processing, this is a rigorous step, since, in case of isolation of DNA or viral RNA representative of a community, it becomes complex, due to the lower proportion of viral genomes and the presence of “contaminating” nucleic acids from bacteria and eukaryotes. Therefore, the extraction of genetic material from other microorganisms is essential [89].

The method of DNA sequencing for the older generation was developed in 1970. Sanger et al. [90] developed a methodology that sequenced DNA through DNA polymerase [91]. This method has been improved from the use of deoxyribonucleic acid inhibitors of chain termination [92] and authors report a DNA sequencing based on chemical modification and specific splitting [93]. The shotgun method was developed using the principles of both methods [53].

Among the technologies of sequencing of new generation genetic material most sought after in the market, the technique developed by American Illumina Inc., San Diego, CA, USA, a leader in the segment of genetic decoding, began its commercialization in 2007; in this technique, the sequencing is attributed by DNA amplification and nucleotide terminators marked by fluorophores [94].

Illumina sequencing has an important function, which is the construction of libraries, obtaining the following main objectives: generate fragments of appropriate size; add adapters to the ends of each fragment; and synthesize sample markers (index or barcode). This division can be carried out through enzymatic digestion, mechanical digestion, or polymerase chain reaction (PCR). Another benefit of NGS in relation to previous sequencing techniques is that NGS can provide various data, i.e., to sequence more nucleotides in a single race, which favors a low-cost and high-yielding technique, with the purpose of obtaining data in different forms [50,54].

One study defined the use of microscopic identification as the gold standard for analyzing rumen protozoa [77]. Consequently, techniques such as this require a high level of experience of the researcher, as well as the strict following of criteria [78], but the microscopic technique has several advantages when compared with the molecular methods of PCR. In electron microscopy, the variation in the number of copies of RNA genes between different genes and different growth conditions can distort the observed concentration [49].

### Future Prospects

The future of rumen microbiology analysis is intrinsically linked to the advancement of next-generation sequencing (NGS) technologies and metagenomics methods. As these technologies continue to evolve, they provide a more comprehensive and detailed view of the microbial community in the rumen of ruminants.

The use of NGS techniques allows the identification and characterization of microorganisms with unprecedented resolution, providing more accurate data on microbial diversity, their functions, and their interactions. In addition, the integration of multi-omics approaches, such as metatranscriptomics and metaproteomics, offers a deeper understanding of the metabolic activities and gene expressions of rumen microorganisms. These emerging technologies not only expand our understanding of the complexity of the rumen ecosystem, but also have the potential to identify predictive biomarkers of animal performance, health, and feed efficiency.

To understand the current gaps in the rumen ecosystem, it is important to develop new studies that aim to investigate the interaction between rumen microorganisms and their environment. Thus, more detailed studies should be carried out on the effects of nutritional factors, such as different types of feed and supplements, on the composition and function of the rumen microbiota. In addition, the mechanisms underlying the interactions between microorganisms and the impact of these interactions on animal health and performance should be further investigated.

One promising field of study involves the discovery of specific biomarkers linked to animal performance and feed efficiency. These biomarkers have the potential to revolutionize livestock production, allowing for personalized diets and management strategies. To fully understand the metabolic pathways and adaptive responses of the rumen microbiota to environmental and dietary changes, it is crucial to integrate multiomic approaches, such as metatranscriptomics and metaproteomics, into longitudinal studies and controlled experiments. By doing so, we can gain a deeper understanding of how the rumen microbiota affects food digestion, nutrient metabolism, and host health. Ultimately, this knowledge will drive significant advances in sustainable animal production and nutrition.

## 6. Conclusions

The following key points can be drawn from this review:

1. An analysis of the literature on rumen microbiology in ruminants in different countries demonstrated the complexity of microbial interactions in rumen. As observed variations in microbial profiles, resulting from various environmental factors, it is highlighted the need for nutritional evaluation and production of ruminants, considering as specific conditions of each region. An in-depth understanding of these microbiological adaptations provides a solid basis for the development of management strategies aimed at optimizing animal health and performance in diverse contexts.
2. In the ruminal microbiota, the genera of protozoa and fungi most evidenced in studies using ruminal fluid were *Entodinium* spp. and *Aspergillus* spp., respectively, and *Fibrobacter* spp. genus for bacteria.
3. About the techniques used, it can be seen that DNA extraction, amplification, and sequencing were the most cited in the studies evaluated. Therefore, this review describes what is present in the literature and provides an overview of the main microbial agents in the rumen and the molecular techniques used.
4. In addition, the review addressed the importance of ongoing research in the field of rumen microbiology, highlighting the need for more in-depth research to elucidate the precise mechanisms underlying microbial responses to different environments. Advanced knowledge of these interactions can potentially inform more sustainable agricultural practices and more efficient animal feeding strategies, helping to mitigate environmental challenges and optimize livestock production on a global scale.

In this context, elucidating the diversity and complexity of microbial interactions in the rumen of ruminants shows that environmental factors have a substantial influence on both the composition and function of these microbial communities. In this field, it is important to carry out further studies to deepen our understanding of the mechanisms by which microorganisms respond to different environments in more detail.

This new insight observed in the review can enlighten us on how microorganisms adapt to environments or conditions and help develop better management techniques to deal with animal health and performance quality issues that are affected by certain situations. Thus, these microbial interactions will guide the scientific community towards sustainable agricultural practices and effective animal feeding strategies, contributing significantly to reducing environmental challenges and maximizing animal production worldwide.

## Figures and Tables

**Table 1:** Information on the articles used in the review.

Year	Authors	Title	Species	Breed	NA	Diet
2008	Martinele et al. [20]	Ciliated protozoa in the rumen of cattle fed elephant grass diets with two levels of concentrate ^+^	Cattle	Mestizo	7	*Elephant grass*
2008	Oyleke and Okusanmi [21]	Isolation and characterization of cellulose hydrolysing microorganism from the rumen of ruminants	Sheep, goats, and cattle	^Ῠ^	5	^ῨῨῨῨ^
2009	Rispoli et al. [22]	Ciliated protozoa in the rumen of cattle and buffaloes fed diets supplemented with monensin or propolis ^+^	Cattle and buffalo	Holstein e Murrah	8	Corn silage and concentrates based on different products
2012	Jami et al. [23]	Composition and similarity of bovine rumen microbiota across individual animals	Cattle	Holstein	16	30% roughage and 70% concentrate ^ῨῨ^
2012	Almeida et al. [24]	Aerobic fungi in the rumen fluid from dairy cattle fed different sources of forage	Cows and calves	Breed	30	53 kg sorghum/animal; 5 kg concentrate/animal; voluminous *Brachiaria brizantha*
2012b	Tymensen et al. [25]	Structures of free-living and protozoa-associated methanogen communities in the bovine rumen differ according to comparative analysis of 16S rRNA and mcrA genes	Cattle	Black Angus	4	Grass hay and different grains with vitamin supplementation and mineral salt
2013	Jami et al. [26]	Exploring the bovine rumen bacterial community from birth to adulthood	Cattle	Holstein	10	Silage and concentrate ^ῨῨ^
2014	Belanche et al. [27]	Study of methanogen communities associated with different rumen protozoal populations	Sheep	Texel	4	67% ryegrass hay and 33% ground barley
2014	Silva et al. [28]	Rumen protozoa of beef steers raised on tropical pasture during the dry period ^+^	Cattle	Nelore	36	*Brachiaria decumbens* and mineral salt
2014a	Almeida et al. [29]	Cellulolytic activity of aerobic fungi isolated from the rumen of dairy cattle fed tropical forages ^+^	Cows	Holstein	85	*Brachiaria Brizantha*
2015	Morgavi et al. [30]	Rumen microbial communities influence metabolic phenotypes in lambs	Sheep	^Ῠ^	8	Milk replacer, hay and concentrate
2015	Belanche et al. [31]	Effect of progressive inoculation of fauna-free sheep with holotrich protozoa and total-fauna on rumen fermentation, microbial diversity and methane emissions	Sheep	Mestizo	8	Mixed ryegrass and white clover pasture
2016	Abrar et al. [32]	Diversity and fluctuation in ciliate protozoan population in the rumen cattle	Cattle	Holstein and Japonese Black Cattle	3	Concentrate ^ῨῨ^
2017	Danielsson et al. [33]	Methane production in dairy cows correlates with rumen methanogenic and bacterial community structure	Cattle	Red Swedes and Holstein	73	Concentrate and silage based on different products
2017	Nigri et al. [34]	Rumen protozoa population in zebu steers fed with or without roughage ^+^	Cattle	Nelore	50	*Brachiaria* spp. and mineral supplementation
2018	Khiaosa et al. [35]	Factors related to variation in the susceptibility to subacute ruminal acidosis in early lactating Simmental cows fed the same grain-rich diet	Cattle	Simmental	18	Concentrate: 20–60% depending on the group ^ῨῨ^
2018	Neubauer et al. [36]	Differences between pH of indwelling sensors and the pH of fluid and solid phase in the rumen of dairy cows fed varying concentrate levels	Cattle	Holstein	8	Grass silage and concentrate ^ῨῨ^
2018	Iqbal et al. [37]	Comparative study of rumen fermentation and microbial community differences between water buffalo and Jersey cows un-der similar feeding conditions.	Buffalo and cattle	Jersey	8	Corn silage and concentrates based on different products
2018	Duarte et al. [38]	Anaerobic fungi in the rumen of heifers and dairy cows fed different tropical roughages	Cattle	Mestizo	100	*Brachiaria* spp.
2019	Jesus et al. [39]	Characterization of ruminal bacteria in grazing Nellore steers	Cattle	Nelore	3	70% Tifton 85 roughage and 30% concentrate based on different products
2019	Souza et al. [40]	Molecular detection of fermentative bacteria groups in the rumen of cattle and buffalo in Santarém-PA ^+^	Buffalo and cattle	^Ῠ^	10	^ῨῨ^
2019	Luna et al. [41]	Isolation, biochemical characterization, and phylogeny of a cellulosedegrading ruminal bacterium	Cattle	Holstein	^ῨῨῨ^	Pasture of *Lolium perene* L.
2019	Dong et al. [42]	Weaning methods affect ruminal methanogenic archaea composition and diversity in Holstein calves	Cattle	Holstein	6	Nutritional composition produced by the group
2020	Zhang et al. [43]	Effect of high-concentrate diets on microbial composition, function, and the VFAs formation process in the rumen of dairy cows	Cattle	Holstein	4	Concentrate: 40–70% depending on the group ^ῨῨ^
2020	Chen et al. [44]	Effects of soybean lecithin supplementation on growth performance, serum metabolites, ruminal fermentation and microbial flora of beef steers	Cattle	Simmental	60	Soy lecithin and dry matter ^ῨῨ^
2020	Freitas et al. [45]	Microbial patterns in rumen are associated with gain of weight in beef cattle	Cattle	Braford	17	12 kg of forage and native pasture
2021	Alves et al. [46]	Rumen bacterial diversity in relation to nitrogen retention in beef cattle	Cattle	Nelore	8	Protein concentrate and sugar cane
2024	Lima et al. [47]	Rumen bacterial diversity in relation to nitrogen retention in beef cattle	Cattle	Nelore	4	T1, no additive (CON); T2, inclusion of 90 g of sodium bicarbonate (BIC); T3, inclusion of 90 g of L. calcareum (L90); and T4, inclusion of 45 g of L. caldarium (L45).

Note: ^+^ Title in another language and translated into English. ^Ῠ^ does not show breed; ^ῨῨ^ does not specify type of forage; ^ῨῨῨ^ does not show how many animals were used; ^ῨῨῨῨ^ diet used not specified. NA—number of animals.

**Table 2:** Information on articles according to year, authors, title, and molecular methods used to identify rumen microbiology.

Year	Authors	Title	Method
2020	Palevich et al. [48]	Complete genome sequence of the polysaccharide-degrading rumen bacterium Pseudobutyrivibrio xylanivorans MA3014 reveals an incomplete glycolytic pathway	DNA sequencing
2010	Medinger et al. [49]	Diversity in a hidden world: potential and limitation of next-generation sequencing for surveys of molecular diversity of eukaryotic microorganisms	DNA amplification and sequencing
2011	Elshire et al. [50]	A robust, simple genotyping-by-sequencing (GBS) approach for high diversity species.	DNA genotyping and sequencing by restriction enzymes (REs)
2012	McSweeney et al. [51]	Commission on genetic resources for food and agriculture. Microorganisms and ruminant digestion: State of knowledge, trends and future prospects.	DNA extraction and quantitative real-time PCR
2012	Singh et al. [52]	Metagenomic analysis of Surti buffalo (Bubalus bubalis) rumen: a preliminary study.	DNA extraction and sequencing
2018	Morey et al. [53]	High throughput DNA sequencing: the new sequencing revolution.	DNA amplification and nucleotide terminators marked by fluorophores
2020	Hess et al. [54]	DNA extraction method in rumen microbiology studies	16S rRNA gene sequencing and DNA extraction

Note: DNA—deoxyribonucleic acid. PCR—polymerase chain reaction.

## Data Availability

The data presented in this study are available upon reasonable request from the corresponding author.
